# What can we learn from the COVID-19 pandemic? Resilience for the future and neuropsychopedagogical insights

**DOI:** 10.3389/fpsyg.2022.993991

**Published:** 2022-09-12

**Authors:** Patrizio Paoletti, Tania Di Giuseppe, Carmela Lillo, Tal Dotan Ben-Soussan, Aras Bozkurt, Golnaz Tabibnia, Kaltrina Kelmendi, Gaye Watson Warthe, Rotem Leshem, Vinca Bigo, Anthony Ireri, Cecilia Mwangi, Nandan Bhattacharya, Giulia Federica Perasso

**Affiliations:** ^1^Istituto di Ricerca in Neuroscienze, Educazione e Didattica, Fondazione Patrizio Paoletti, Assisi, Italy; ^2^Distance Education Department, Anadolu University, Eskişehir, Turkey; ^3^Department of Psychological Science, University of California, Irvine, Irvine, CA, United States; ^4^Department of Psychology, University of Prishtina, Hasan Prishtina, Pristine, Kosovo; ^5^Faculty of Health, Community and Education, Mount Royal University, Calgary, AB, Canada; ^6^Department of Criminology, Faculty of Social Sciences, Bar-Ilan University, Ramat Gan, Israel; ^7^Kedge Business School, Talence, France; ^8^Department of Educational Psychology, Kenyatta University, Nairobi, Kenya; ^9^UGC-Human Resource Development Centre, Jadavpur University, Kolkata, West Bengal, India

**Keywords:** resilience, COVID-19, brain functioning, social interconnectedness, education, neuropsychopedagogical, self-training

## Introduction

### The psychological effects of COVID-19 pandemic

The COVID-19 pandemic caused a spike in the prevalence of stress and anxiety (Yao et al., 2020). Globally, between January 2020 and January 2021, depressive disorders increased by 27% while anxiety disorders increased by 25% (Santomauro et al., 2021). Despite attempts to promote mental health practices through digital resources (Arenliu et al., 2020), the pandemic massively impacted adolescents and young adults as it doubled the prevalence of depressive and anxiety symptoms among youths (Racine et al., 2021).

A “new normality” has changed our everyday life (Bozkurt and Sharma, 2020) and social norms (Andriani, 2020). Previous research on natural disaster preparedness (Kostouros and Warthe, 2020; Warthe et al., 2022; Di Giuseppe et al., 2023 (In Press)) does not suggest how to deal with a long-term phenomenon such as a pandemic that has caught global communities unprepared. COVID-19 and its effects have highlighted the urgency for individuals and societies to fortify themselves at a psychosocial level.

A key construct to address this need is resilience, defined as the ability to adaptively cope with adversity (Luthar et al., 2000), resulting from the dynamic interaction among genetic, biological, and environmental factors (Herrman et al., 2011). In this paper, we refer to resilience as the process that allows the individual, group, and community to cope with, overcome, and emerge strengthened from negative experiences (Grotberg, 1995; Hamby et al., 2018). To date, most studies on the effects of the pandemic primarily examine the risk factors for individuals' health, while studies that explore resources and assets that promote positive coping with the adverse effects of the pandemic are still scarce.

Starting from this premise, the following paper is the result of a collective reflection shared by resilience researchers across the globe (Fondazione Patrizio Paoletti, 2022), inquiring about how the pandemic can be considered a catalyst for change for building more resilient communities and social structures. As a contribution to facing the current emergency, resilience researchers share four interdisciplinary insights that are presented in the current paper.

## Discussion

### What can we learn from COVID-19?

#### Insight 1: Being more aware of brain functioning and its potential can help us face the global increase in anxiety and depression

Every individual, community, and institution should access theoretical and practical knowledge about the human brain and mental functioning to become more resilient. Adults can become more resilient by reprogramming their brains to counteract the effects of stress by enhancing the activity of the prefrontal cortex and coping circuits (Fredrickson, 1998; Davidson, 2000; Korb, 2015; Tabibnia and Radecki, 2018). Stressors negatively impact the prefrontal cortex, which is the seat of higher functions (Diamond, 2016), and the synthesis of brain-derived neurotrophic factor (BDNF), which is critical for neuronal plasticity (Fritsch et al., 2010). Tabibnia (2020) highlights three neuroscience-based strategies that are key for building resilience: (1) reducing the negative impact of experience (e.g., *via* cognitive reappraisal) and therefore the distress responses of the amygdala, hypothalamic-pituitary-adrenal axis, and autonomic nervous system; (2) increasing positive experience (e.g., optimism and social cohesion) and associated activation of mesostriatal reward circuits, which can counteract stress responses; (3) cultivating self-transcendence (with techniques such as meditation, mindfulness), thereby reducing the activation of the default mode network (DMN) and associated rumination, self-reflection, and mind-wandering. Different meditation techniques, such as Quadrato Motor Training (QMT), can indirectly incentivize resilience by increasing prefrontal activation and decreasing the DMN (Dotan Ben-Soussan et al., 2013; Paoletti and Ben-Soussan, 2021; Paoletti et al., 2022a).

#### Insight 2: It is necessary to develop an awareness of human interconnectedness to overcome adversity

Our wellbeing is not only dependent on our actions toward ourselves and toward others but is also influenced by the actions of others (White and McCallum, 2021). We can be more resilient when we are focused on other individuals and their needs through empathy, compassion, and tolerance (Jordan, 2004; Slavich et al., 2021). Additionally, the support of family, friends, and community is considered another source of strengths crucial to developing resilience and overcoming adversity in diverse cultural contexts and populations (Hamby et al., 2018; Kelmendi and Hamby, 2022). Conversely, “self-centered” resilience reinforces social alienation, hindering the development of a sense of brotherhood and responsibility to others (Mahdiani and Ungar, 2021). Individuals' sense of community is not yet sufficiently developed to cope with a global emergency (Hirsch, 2010; Turcotte and Caron, 2017), as indicated by the social tensions about biopower (Arminjon and Marion-Veyron, 2021) and the difficulties in complying with anti-contagion measures (Hills and Eraso, 2021). Noticeably, activities such as artistic engagement can improve the level of resilience of individuals and communities (Reed et al., 2020). The creative process (e.g., drawing) allows the expression and processing of emotions within group interventions as well as with COVID-19 patients (Bhattacharya et al., 2022). Plus, it has been demonstrated that the creative potential represents a protective factor against COVID-19 lockdown stress (D'Anselmo et al., 2022).

#### Insight 3: School-programs should educate next generations in resilience

To face the global spread of anxiety and depression among youth as a consequence of the pandemic (Racine et al., 2021), resilience must be promoted and integrated in educational settings. The educational landscape should create personalized and collective support strategies and integrate resilience in educational curricula around the world (Mwangi et al., 2017; Kelmendi and Hamby, 2022). Educational systems must develop new strategies to survive the long-term crisis sustainably, ensuring that no one is left behind (Bozkurt, 2022) in light of socioeconomic disparities between countries. Fundamental to this is the digitalization of educational systems, possibly facilitated by preparatory interventions to support students, teachers and parents (Hyseni- Duraku and Hoxha, 2020).

#### Insight 4: Self-training resilience tools can allow individuals, groups, and communities to access neuro-psycho-pedagogical knowledge to face adversities, uncertainty, and changes in everyday life

Self-educational programs can help people to understand the links between neuro-psycho-physiological states, emotions, and behaviors to re-interpret events and emergencies and create a resilient community. In this sense, Patrizio Paoletti Foundation designed a self-administered resilience-training program entitled “Ten Keys (i.e., suggestions) for Resilience” (Table 1; Paoletti et al., 2022a). The program was born in the framework of the Sphere Model of Consciousness (Paoletti, 2002a,b; Paoletti et al., 2022c; Paoletti and Ben Soussan, 2019), and was inspired by interdisciplinary literature (Fredrickson, 1998; Davidson, 2000; Korb, 2015; Paoletti, 2018; Tabibnia and Radecki, 2018). The 10 Keys for resilience are theoretical-practical indications describing how a resilient brain works. These indications support the process of awareness through the proactive re-signification of experience at a cognitive level, improving emotional regulation and the physical wellbeing of the individual. Taken collectively, the 10 Keys create an expanded conceptualization of resilience, where the individual trains to overcome adversity to transform the adversity into an opportunity for personal and collective growth (Paoletti et al., 2022a). The 10-Keys are organized according to the four neuro—psycho-pedagogical principles of the Pedagogy for the Third Millennium (PTM; Paoletti, 2008, 2018): Observation, Mediation, Translation, Normalization Paoletti and Selvaggio, 2011a,b, 2012, 2013; Paoletti et al., 2022a. The Ten-Keys were applied in emergency and challenging context (e.g., earthquake survivors, inmates, and juvenile penal justice educators) even during COVID-19 pandemic (Di Giuseppe et al., 2022c; Di Giuseppe, 2022; Di Giuseppe et al., 2022a,b, 2023 (In Press); Maculan et al., 2022; Paoletti et al., 2022b).

**Table 1:** Ten-Keys for resilience by Patrizio Paoletti Foundation. Revised version from (Di Giuseppe et al., 2022a).

**Key**	**Content**	**Neuropsychopedagogical principle**
(1) Focus on what you can control and make small decisions.	Bringing attention to the *here and now* and making small decisions to overcome uncertainty.	Observation and Self-observation (Paoletti and Selvaggio, 2011a)
(2) Identify an attainable, exciting, measurable goal.	Setting goals, foreseeing obstacles, and cultivating positive beliefs.	Observation and Self-observation (Paoletti and Selvaggio, 2011a).
(3) Several times a day become aware of your posture.	Setting body posture for physical activation, raising attention and self-confidence.	Observation and Self-observation (Paoletti and Selvaggio, 2011a).
(4) Be inspired by stories.	Following resilience role-models.	Mediation (Paoletti and Selvaggio, 2011b).
(5) Ask yourself what is really important.	Training in self-motivation, listening to your most intimate preferences.	Mediation (Paoletti and Selvaggio, 2011b).
(6) Cultivate gratitude.	Learning to cultivate positive emotions (e.g., gratitude) and to manage negative emotions.	Mediation (Paoletti and Selvaggio, 2011b).
(7) Appreciate the other as a resource, cultivate and expand your social network.	Listening, sharing experiences, enhancing social and interpersonal resources.	Translation (Paoletti and Selvaggio, 2012).
(8) Cultivate curiosity.	Learning from everything and from every experience.	Translation (Paoletti and Selvaggio, 2012).
(9) Practice a few minutes of silence.	Practicing multiple times a day intentional silence, envisioning the best version of yourself.	Normalization (Paoletti and Selvaggio, 2013)
(10) Embrace and transform: before bedtime, generate your tomorrow today.	Self-programming and foreshadowing of the future through proactive storytelling of daily life.	Normalization (Paoletti and Selvaggio, 2013)

## Conclusion

The four insights, resulting from the encounter among resilience researchers (Fondazione Patrizio Paoletti, 2022), offer an interpretation of the experiences lived during the pandemic. Taken collectively, these insights integrate neuroscience, psychology, and pedagogy to face the post-pandemic world with an interdisciplinary knowledge. The insights aim at helping us to face the current emergency and preparing us for the future, overcoming the global spread of anxiety and stress highlighted by scientific literature. Next generations could benefit from re-analyzing the concept of resilience as a set of strengths of the individual and community to promote wellbeing and mental health. It is also crucial, in the future of the educational systems, to integrate resilience in school programmes, and to promote self-training in resilience across the life cycle. These neuro-psycho-pedagogical insights eventually can be translated to a wide range of emerging future challenging situations which we are currently facing, such as financial instability, war, and climate change.

## Author contributions

PP conceived and supervised the realization of the manuscript in the structure. TD, CL, GP, PP, AB, GT, KK, GW, VB, RL, TB-S, AI, CM, and NB contributed in the writing of the insights presented in the article. All authors contributed to the revision of the article and approved the final version.

## Conflict of interest

The authors declare that the research was conducted in the absence of any commercial or financial relationships that could be construed as a potential conflict of interest.

## Publisher's note

All claims expressed in this article are solely those of the authors and do not necessarily represent those of their affiliated organizations, or those of the publisher, the editors and the reviewers. Any product that may be evaluated in this article, or claim that may be made by its manufacturer, is not guaranteed or endorsed by the publisher.
